# Plant root plasticity during drought and recovery: What do we know and where to go?

**DOI:** 10.3389/fpls.2023.1084355

**Published:** 2023-03-16

**Authors:** Congcong Zheng, Helena Bochmann, Zhaogang Liu, Josefine Kant, Silvia D. Schrey, Tobias Wojciechowski, Johannes Auke Postma

**Affiliations:** ^1^ Institute of Bio- and Geosciences – Plant Sciences (IBG-2), Forschungszentrum Jülich GmbH, Jülich, Germany; ^2^ Faculty of Agriculture, University of Bonn, Bonn, Germany; ^3^ Key Laboratory of Ecosystem Network Observation and Modeling, Institute of Geographic Sciences and Natural Resources Research, Chinese Academy of Sciences, Beijing, China

**Keywords:** bibliometric analysis (BA), intermittent drought, root dynamics, recovery, nutrient homeostasis

## Abstract

**Aims:**

Drought stress is one of the most limiting factors for agriculture and ecosystem productivity. Climate change exacerbates this threat by inducing increasingly intense and frequent drought events. Root plasticity during both drought and post-drought recovery is regarded as fundamental to understanding plant climate resilience and maximizing production. We mapped the different research areas and trends that focus on the role of roots in plant response to drought and rewatering and asked if important topics were overlooked.

**Methods:**

We performed a comprehensive bibliometric analysis based on journal articles indexed in the Web of Science platform from 1900-2022. We evaluated a) research areas and temporal evolution of keyword frequencies, b) temporal evolution and scientific mapping of the outputs over time, c) trends in the research topics analysis, d) marked journals and citation analysis, and e) competitive countries and dominant institutions to understand the temporal trends of root plasticity during both drought and recovery in the past 120 years.

**Results:**

Plant physiological factors, especially in the aboveground part (such as “photosynthesis”, “gas-exchange”, “abscisic-acid”) in model plants Arabidopsis, crops such as wheat and maize, and trees were found to be the most popular study areas; they were also combined with other abiotic factors such as salinity, nitrogen, and climate change, while dynamic root growth and root system architecture responses received less attention. Co-occurrence network analysis showed that three clusters were classified for the keywords including 1) photosynthesis response; 2) physiological traits tolerance (e.g. abscisic acid); 3) root hydraulic transport. Thematically, themes evolved from classical agricultural and ecological research *via* molecular physiology to root plasticity during drought and recovery. The most productive (number of publications) and cited countries and institutions were situated on drylands in the USA, China, and Australia. In the past decades, scientists approached the topic mostly from a soil-plant hydraulic perspective and strongly focused on aboveground physiological regulation, whereas the actual belowground processes seemed to have been the elephant in the room. There is a strong need for better investigation into root and rhizosphere traits during drought and recovery using novel root phenotyping methods and mathematical modeling.

## 1 Introduction

Drought undoubtedly represents the most serious hazard to livestock and crops in nearly every part of the world; an estimated 55 million people are affected by droughts globally every year (UNDRR, 2021). Both historical records and model simulation results suggest the increased risk of drought in the twenty-first century will happen *via* either decreased precipitation and/or increased evaporation (Dai, 2013; Langenbrunner, 2021). Globally, more frequent and intense drought events are expected to occur, particularly in arid and semiarid regions (Davidowitz, 2002; Spinoni et al., 2014; Touma et al., 2015). Regions with high seasonable variability will become even more variable and experience more extreme weather events (Konapala et al., 2020). Global synthesis analysis predicted that when water supply decreases by approximately 40%, wheat and maize yields will reduce by 21% and 39%, respectively (Daryanto et al., 2016). However, in natural and agricultural ecosystems, short- and long-term droughts happen frequently but are usually not permanent and the plants can continue to grow or even grow faster during the later recovery or rewatering period. IPCC defines drought as “a period of abnormally dry weather long enough to cause a serious hydrological imbalance” (Masson-Delmotte et al., 2018). In accordance with the IPCC drought definition, we suggest defining plant recovery from drought as “the period after a drought during which the hydrological balance is restored”. Since drought can cause lasting damage, recovery might not be 100% compared to well-watered conditions and the definition of “restored” is the point where no significant improvement is further observed, despite sufficient water supply. Considering the growing population, and climate change, a better understanding of plant response during drought and recovery offers the potential to increase plant climate resilience and production.

Water availability limits plant growth and final production nearly in all natural ecosystems, this is especially true in agriculture ecosystems. As a fundamental aspect of plant adaptability and yield, the role of root plasticity in drought tolerance has received increasing attention in recent years (Lynch, 1995; Comas et al., 2013; Kashiwagi et al., 2015; Gao and Lynch, 2016; Koevoets et al., 2016). At the same time, root systems are key to plant growth, water uptake, water perception, and signaling (Lynch, 2007; Hamanishi and Campbell, 2011; Carley et al., 2022). Considerable progress has been made in unraveling the mechanisms of drought responses in plant roots which involve an array of molecular, anatomical, physiological, morphological, and biotic regulations aiming at both tolerance and avoidance of drought stress. For example, during drought plant roots modify aquaporin (AQP) and dehydrin gene expression (Reddy et al., 2017), change metaxylem vessel diameter, root diameter, and crown root number (Gao and Lynch, 2016; De Bauw et al., 2019; Klein et al., 2020), increase ABA levels and change carbon allocation (Zhang et al., 2006; Guo et al., 2021), and alter root microbiota composition (Santos-Medellin et al., 2021). The belowground plasticity is accompanied by aboveground responses, like ABA production in the shoot and aquaporin contributed stomatal closure which are, however, not the focus of this review. During the subsequent recovery period after drought, the hydrological balance restored in plants and soil makes these changes return to normal (e.g. comparable to well-watered conditions), e.g., decrease ABA level, and restored fine roots through root regrowth (Lauenroth et al., 1987; Luo, 2010; Fang and Xiong, 2015; Maurel and Nacry, 2020). Although both drought resistance and post-drought recovery are key determinants of plant growth, some recent studies suggest that recovery may play a more significant role in plant drought adaptation than drought resistance itself (Chen et al., 2016; Gonzalez-Hernandez et al., 2021). Thus, to increase plant resistance and resilience [For definitions see (Enright et al., 2014; Hoover et al., 2021)], and maximize plant production, understanding root plasticity during both drought and recovery is necessary (Vilonen et al., 2022). A comprehensive mechanistic understanding of relevant processes during drought recovery is, however, lacking.

**Figure 1:**
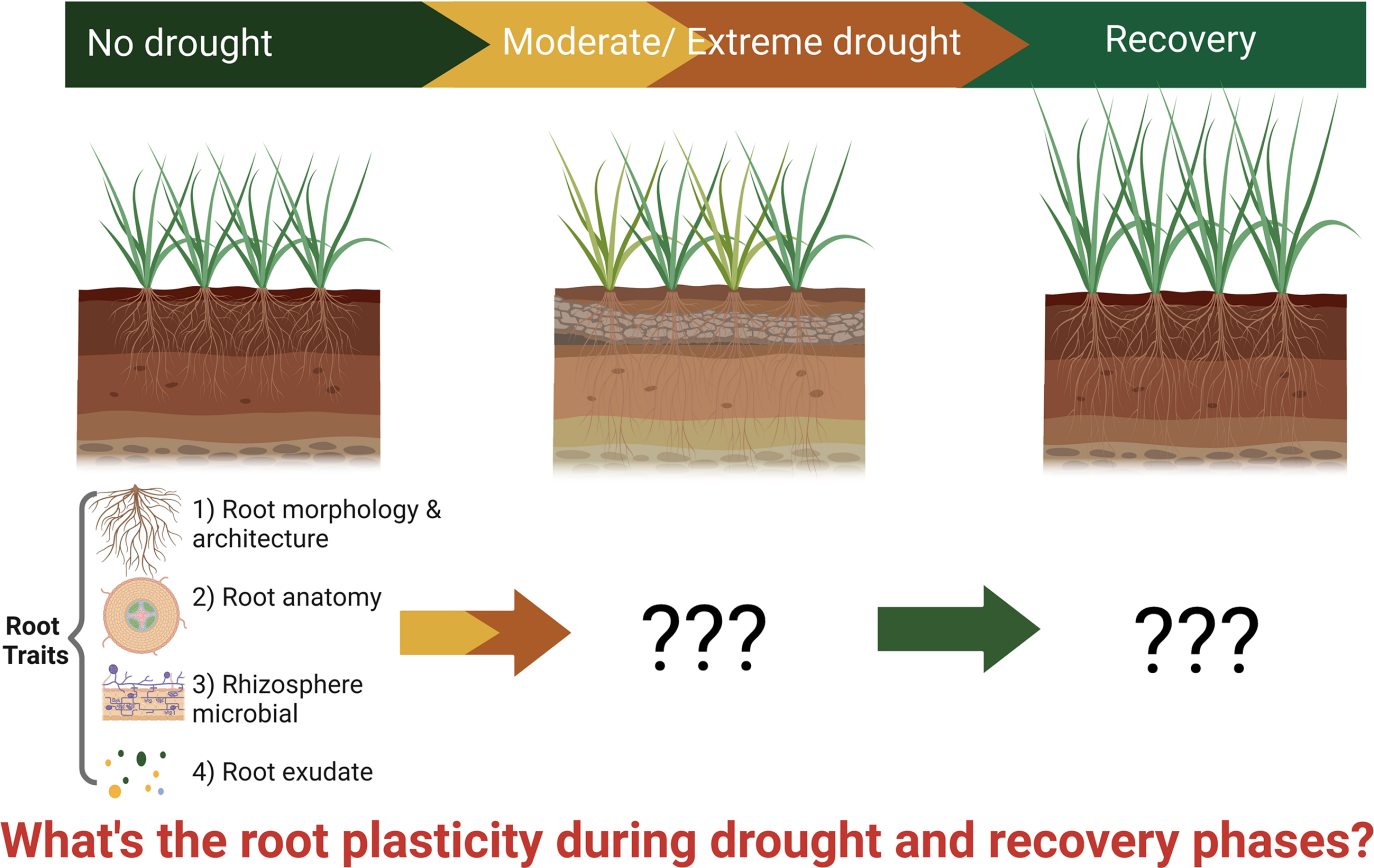
Schematic representation of root plasticity during drought and recovery. The image is created with BioRender. Created with BioRender.com.

Bibliometric analysis is an effective tool to describe the knowledge status, features, and trends in a certain discipline and is increasingly used to summarize the literature using objective statistics. Specifically, bibliometrics can clarify the current progress of a certain research field and show the temporal trends of research disciplines and research hotspots (Aria and Cuccurullo, 2017). It includes qualitative and quantitative analysis of publications indexed by databases based on statistics and computing technology, which makes the outputs more objective and reliable (van Eck and Waltman, 2014). After Alan Pritchard proposed the bibliometrics method in 1969, more scientists use this approach to review the subject’s progress, which provides a comprehensive evaluation at various levels; current reviews in nitrogen deposition and soil phosphorus fractions are good illustrations (Pritchard, 1969; Oliveira Filho and Pereira, 2020; Li et al., 2022). To review the entire landscape of root plasticity, including root morphology/architecture, anatomy, exudation and rhizosphere microbiomes during drought and recovery like shown in **Figure 1**, we conducted a comprehensive bibliometric analysis. The objectives of this study include a) understanding the research patterns of root plasticity during drought and recovery research globally, b) developing an accurate overview of the scientific output of root plasticity during drought and recovery over time and space, and c) providing potential research trends and hotspots for future studies.

## 2 Materials and methods

Bibliometric data collection was carried out on 29 December 2021 based on the Science Citation Index-Expanded (SCI-E) database in the “Web of Science Core Collection” (http://www.webofknowledge.com), considering the SCI-E database could provide comprehensive coverage of the most important publications over the world and include also explicit reference details which enable us to track the intellectual progress trend of our focused topic. Only one database, “Web of Science Core Collection”, was used because it is currently not possible to conduct the bibliometric analysis on merged databases. We searched for publication topics with the following search command: (“Root”) AND (“Drought” OR “Water stress” OR “Water deficit” OR “Water scarcity”) AND (“Recovery” OR “Rewater” OR “Rewet” OR “Legacy effect”) NOT (“Submergence” OR “Waterlogging” OR “Flood”). The terms “AND” and “OR” were used to maximize the correct selection of interest articles, e.g., the term “AND” was used to enable the research for all terms of root plasticity during both drought and recovery, the term “OR” was used to search for at least one of the terms, and the term “NOT” was used to exclude irrelevant research which appeared in water stress and recovery. Thus, a total of 1102 publications were obtained for all years of publication through Dec. 2021. Publications were screened to ensure main information was included such as title, authors, keywords, ISO source abbreviation, abstract, publication year, volume, and issue, resulting in 1086 proper records (more details can be found in **Table 1**). Besides “Web of Science Core Collection” database, we also queried the “Scopus” database. Compared with the “Scopus” database, “Web of Science Core Collection” database identified a greater number of publications (1086 *vs* 880) with more than 80% overlap with the “Scopus” database. We concluded that a more complete result was obtained with the “Web of Science Core Collection” database (For example top-cited publications results in **Table S1**). Data were then downloaded and converted into a BibTex format for further bibliometric analyses in R (Bibliometrix package in R software).

**Table 1:** Main information in relation to root plasticity during drought and recovery collection.

Description	Data
Timespan	1975–2022
Sources (Journals, Books, etc)	334
Publications	1086
Author’s Keywords (DE)	3134
Authors	4112
Average years from publication	10.1
Average citations per publication	34.99
Author Appearances	5091
Authors per publication	3.79
Collaboration Index	3.85

Collaboration index=Authors of multi-authored publications/Multi authored publications.

We first analyzed a) the number of publications per year and b) the number of scientific productions per country and institute. To better understand the distribution of the output in different journals, we computed the article numbers, the number of citations, and the journal’s topical *h*-index. Note that the *h*-index was based on citations acquired in the WoS Core Collection and were different from those published by other databases, notably Google Scholar or Scopus (Hirsch, 2005; Oliveira Filho and Pereira, 2020).

To further investigate trends and advances of the focused topic, keywords frequency and relationship analysis were carried out with the word cloud and co-occurrence analysis. To make the frequency analysis more precise, we merged the common words used in all publications from plural/singular, Latin plant names/common name to the singular and common one firstly, e.g., plants-plant, roots-root, leaves-leaf, *Arabidopsis-thaliana*-Arabidopsis, *Zea-mays*-maize, and *Oryza-sativa*-rice. We used the word cloud to identify the 50 most frequent keywords used in “root plasticity during both drought and recovery research” over the past 120 years. We further drew the keywords co-occurrence network with the 50 most popular keywords to determine the latest research hotspots in root plasticity during the drought and recovery topics. Here, different circle sizes represent keywords’ frequency appearance in a cluster; different colors depict different clusters, indicating that these keywords are likely to appear in the same publication. The lines connecting the circles represent the co-occurrence of keywords, with thicker lines, representing stronger relations. To better understand the temporal evolution of research topics, a temporal trend analysis of keywords was carried out and divided the publications of 1975-2022 into four periods (1975–1995, 1996–2005, 2006–2015, and 2016–2022). By dividing the timespan into time slices, the evolution of topics in a specific research field can be shown by the alluvial graph (Aria and Cuccurullo, 2017). Besides, by applying a clustering algorithm to a keyword network, we can highlight certain topics of a given field. We mainly analyzed two themes, namely, basic and motor themes. Basic themes are fundamental concepts that haven’t been well-developed. Motor themes represent topics that are both important and well-developed (Cobo et al., 2011). All bibliometric and data analyses, and figures were done with R 4.0.4 and Biorender software.

## 3 Results and discussion

### 3.1 Research areas and temporal evolution of keyword frequencies

#### 3.1.1 Most popular keywords

**Figure 2:**
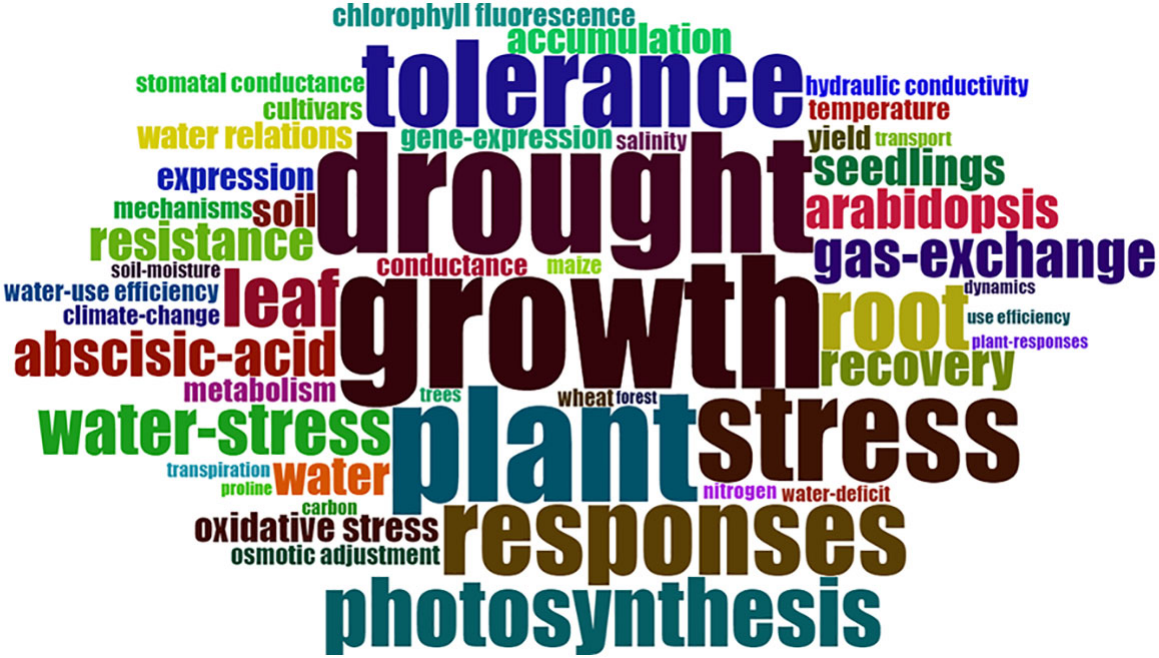
Top 50 keywords represented by the word cloud; labels are usually single words. and the frequency of each label is shown with font size. The biggest word “growth” appeared 266 times with a frequency of 7%, while the smallest word “forest” only showed up 26 times with a frequency of 1%.

Plant “growth” (total frequency of 7%) was the most frequently used keyword (**Figure 2**). This is likely because drought and recovery represent different periods of plant water status and growth connects them and is also frequently used as an indicator of drought and recovery. Additionally, in contrast to the many shoot-only studies that are not part of this analysis, root-related studies usually take a whole-plant approach. When we ignore common words like “stress”, “drought”, “tolerance”, “response”, “plant” and “water-stress”, the high-frequency keywords in root plasticity during both drought and recovery research can be grouped into 3 types: 1) physiological factors: photosynthesis, gas-exchange, abscisic-acid, (stomatal) conductance, osmotic adjustment, accumulation, and transpiration; 2) different plant species: Arabidopsis, wheat, maize, and trees; 3) abiotic factors: temperature, salinity, nitrogen, and climate change (**Figure 2**), which will be discussed below. No words related to biological interactions got into the top 50 list, and the first biological factor “fungi” was found in the top 150 list and appeared only 12 times and thereby had a frequency of 0.2%.

For physiological factors, higher frequency words “photosynthesis”, “gas-exchange”, “stomatal conductance”, and “transpiration” could reflect that aboveground traits received more attention than belowground traits. This shoot-dominated focus continued despite the vital role of roots in determining plant ecology, terrestrial ecosystem functioning, and their designation as the target for the second green revolution (Gašparíková et al., 2002; Lynch, 2007). This is partly because plant water uptake and transport are generally thought to be regulated by the stomata (Li et al., 2020), but probably also due to technological limitations in monitoring root growth dynamics and studying the hydraulic pathways in the root system. Using non-destructive technologies like MRI (Pflugfelder et al., 2017) and SWaP (Dusschoten et al., 2020), a recent study focussing on faba bean and maize has proved that stomatal sensitivity is partly explained by the sensitivity of root hydraulic conductance to soil drying (Müllers et al., 2022b). A better understanding of the role of root conductance in soil drying and rewatering is vital to complete the picture from soil to root to leaf. Associated with the focus on stomatal conductance and morphological leaf traits, “abscisic acid” was another important physiological keyword. For example, Correia et al. (2014) showed that both abscisic acid (ABA) and ABA-glucose ester (ABA-GE) are up-regulated during drought and down-regulated during recovery in the *Eucalyptus globulus*. Besides stomatal closure, ABA controls physiological processes like osmotic regulation, growth inhibition, and transcriptional regulation of stress-responsive gene expression (Zhang et al., 2006; Li et al., 2020).

Of the studied species, the model plant Arabidopsis (total frequency of 81, with a relative frequency of 2%) was the most popular (**Figure 2**). Its popularity is associated with its small size, relatively short lifecycle, ease of growing under low-light lab conditions, and small genome which is instrumental for studying processes at the molecular level. In addition, wheat and maize as important cereals and worldwide staple food, were other popular species. From the first three species, it is clear that herbaceous plants are much more often researched than trees. However, as a group, “trees” still had a relatively high frequency (total frequency of 30, with a relative frequency of 1%). Trees are perennial plants with longer life spans and thus are likely to experience temporal drought, some mortality happens when these plants suffer from hydraulic failure; 2) Tree species generally have thicker stems and large enough vessels than tiny plants which make it easier to monitor water transport non-invasively (Brodribb et al., 2017).

Drought stress strongly interacts with other abiotic stresses. Drought stress is often accompanied by heat stress and is aggravated by salt stress. Salinity also can cause similar problems with drought stress due to the high osmotic potential in the soil, which leads to similar response patterns in plants, consequently, some scientists compare salinity and drought stress effects in their research (Sánchez-Blanco et al., 2014; Koevoets et al., 2016; Ma et al., 2020). Concomitantly, nutrient availability and uptake are inhibited by dry soil (Parrondo et al., 1975; Hira and Singh, 1977; He and Dijkstra, 2014). Climate change may exacerbate these interactions. Although it is challenging to study such interactions, they received relatively much attention as indicated by the frequencies of the words “temperature”, “salinity”, “nitrogen”, and “climate change” (**Figure 2**).

#### 3.1.2 Co-occurrence network of popular keywords

**Figure 3:**
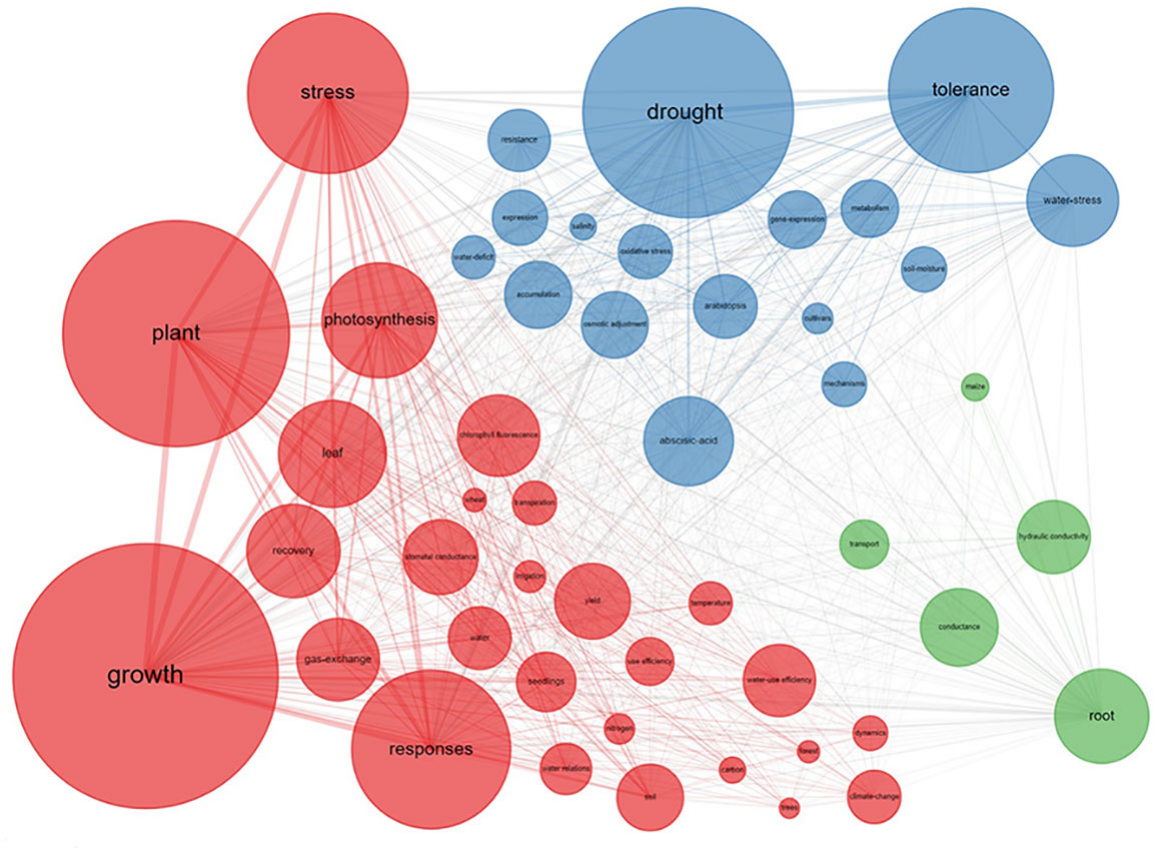
Co-occurrence Network of top 50 keywords. The size of the circle and the connecting lines represent the frequency and the relationship of the keywords, separately. The larger size the rectangular is, the higher the frequency. Similarly, the thicker the line is, the closer the relationship between keywords. Different colors represent different clusters, indicating that these keywords appear more frequently in the same publication. Red cluster, leaf parameters’ response; blue cluster, molecular, and physiological responses; green cluster, root hydraulic and water transport response.

The co-occurrence network of the keywords revealed three clusters which we labeled: “plant growth”, “drought tolerance”, and “root hydraulics” (**Figure 3**). “Growth” was the most popular keyword in the plant growth cluster (i.e., red cluster). Like the word cloud results, this cluster described the plant growth response in association with aboveground traits like “photosynthesis”, “leaf”, “gas exchange”, “chlorophyll fluorescence”, “stomatal conductance”, and “transpiration”. Even though the initial literature search included the keyword “root”, leaf traits had a higher frequency in this cluster, indicating that the study of plant response to drought and recovery is strongly focused on aboveground parameters. The plant growth cluster included words like “trees”, “forest”, and “yield”, hinting at a more agroecological context. For forest ecosystems, climate change has caused more frequent drought events, and scientists have focused on different tree species’ growth under drought and other environmental factors like soil nitrogen deficit and higher temperature. In agricultural ecosystems, wheat production has been the main focus of research. Wheat is known to be deep rooting and relatively tolerant to drought compared to other major grains (Fan et al., 2016).

The plant physiology cluster (blue cluster) contains words associated with (molecular) plant physiology such as “drought”, and “tolerance”, which were the most popular keywords in this cluster and were strongly associated with words like “gene expression”, “osmotic adjustment”, “oxidative stress”, and “abscisic acid” (ABA). In contrast to the “red” cluster, which mainly focuses on plant growth response, the blue cluster mainly focuses on gene and hormone regulation. The keywords in the blue cluster were associated with the model plant Arabidopsis, indicating that molecular physiology is commonly studied in this plant. “Abscisic Acid” bridges back to the “red plant growth” cluster through keywords like “response” and “photosynthesis” (**Figure 3**). Mechanistically, Abscisic Acid regulates the stomatal response and thereby directly influences photosynthesis and growth.

The root hydraulics cluster (green cluster) included “root” as the most popular keyword and its relation to “conductance”, “hydraulic conductivity”, “transport”, and “maize” (**Figure 3**). This cluster comprises research on water transport from the soil through the roots into the shoot. Hydraulic conductance of soil, rhizosphere, roots, and xylem is of great importance to understanding water “transport”. The word “root” was only weakly connected to leaf and physiological traits, possibly indicating a discrepancy in the research. This is surprising, given that plant hydraulic conductance, transpiration, and CO_2_ uptake, are regulated *via* stomatal opening and closure. The ABA-controlled regulation of stomata is, however, sensitive to soil hydraulic properties (Carminati and Javaux, 2020; Abdalla et al., 2021), interacting with the root length and morphology (Müllers et al., 2022a). The mechanisms are still strongly debated (Li et al., 2020). In addition, it seems that root and water-transport researchers have chosen “maize” as their favorite model species. Possibly, because maize has a rather sturdy root system which is more easily studied than the fine roots of Arabidopsis or wheat.

### 3.2 Scientific mapping and trends of the outputs over time

#### 3.2.1 Temporal evolution of the outputs over time

**Figure 4:**
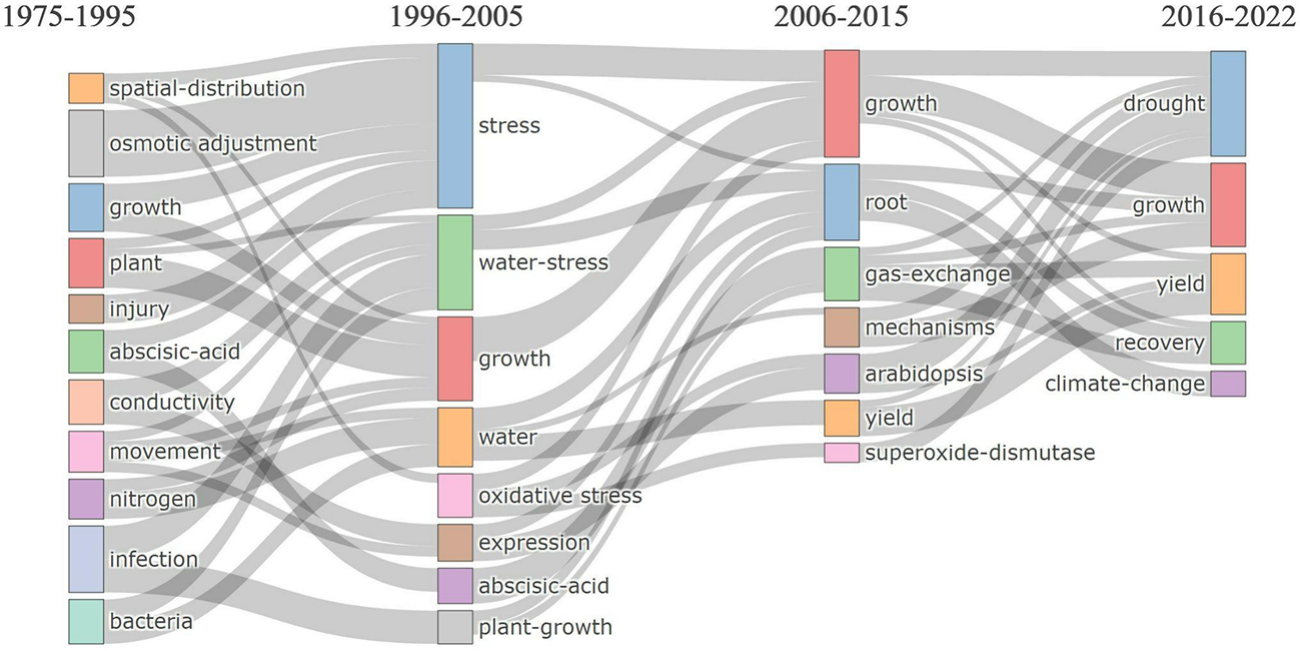
Thematic evolution of popular keywords in regard to root plasticity during drought and recovery research. The horizontal axis represents the time period, and boxes of different colors represent different keywords. The size of each box represents the frequency in different time periods, and the lines between each box reflect the keywords’ temporal evolution, transfer, and inheritance.

The frequently used keywords have changed over the past five decades. From 1975 to 2005, the topics ranged from whole plant physiology to molecular response, but roots were not in focus. Only in recent years, from 2006 to 2022, keywords related to root plasticity to drought and post-drought recovery became more frequent. During this period, the focus shifted to applied aspects such as “yield” and “climate change” as climate change made the need for resilient crop yield more imminent. The keyword “growth” was frequently used in all periods, as it is fundamental to our definitions of drought stress and recovery (**Figure 4**).

During 1975-1995, the main focus was on plant physiology as indicated by the frequent use of words such as “osmotic adjustment”, “growth”, (cellular) “injury”, “abscisic acid” and “conductivity”. Some scientists also investigated the impact of microbes on drought response like endophytic fungi and plant growth-promoting rhizobacteria (PGPR), which explain the appearance of keywords like “infection” and “bacteria” (Ruizlozano et al., 1995). With the advancement of molecular technologies, “expression”, and “oxidative stress” increased from 1996-2005, indicating that more scientists devoted themselves to identifying genes involved in drought tolerance. During 2006-2015, research emphasized mechanisms using model plants and an increasing interest in roots, with frequent keywords: “root”, “gas exchange”, “mechanisms”, “Arabidopsis”, and “superoxide-dismutase”. After 2015, the number of studies increased, and more researchers demonstrated that the recovery period matters to the overall plant performance and that is especially true in the field station under climate change (Hagedorn et al., 2016; Guo et al., 2021; Santos-Medellin et al., 2021). During this period, keywords such as “recovery” and “climate change” were used frequently.

#### 3.2.2 Motor and basic themes

The temporal evolution of keywords’ frequency shows keywords that were gradually used less, like “abscisic acid”, ones that remained stable, like “growth”, and those that gained more attention in recent years, like “climate change” and “yield” (**Figure 4**). Therefore, we added a thematic analysis to further understand the temporal evolution of keywords. The thematic analysis distinguishes “motor” from “basic” themes. Motor themes are both important, well-developed, and highly cited in recent years, whereas basic themes are the main and driving keywords for the research topic but receive a few citations (Cobo et al., 2011; Liu et al., 2021). To the motor themes belonged words like “water stress”, “stomatal conductance”, “abscisic-acid”, “chlorophyll fluorescence”, “biomass”, “proline”, and “hydraulic conductivity” whereas “drought”, “recovery”, “photosynthesis”, “root”, “climate change”, “growth” and “resilience” were the basic themes (**Table 2**). Although “water stress”, “abscisic acid”, “proline” (osmotic adjustment), and “conductivity” were motor themes and important, they were studied intensively in the early stage (**Figure 4**). The root supports growth through water and nutrient uptake, transport, perception, and signaling. Thematic evolution identified oxidative stress and nitrogen as basic themes, well developed in 1975-2005 (**Figure 4**, **Table 2**). In contrast, “recovery” and “root” (note these were part of our initial search terms), and “climate change” had a stronger development during recent years. This is likely to continue in the near future as climate change demands agro-ecological adjustment to the increasing risk of temporal drought.

**Table 2:** Top 10 high-frequency keywords in basic and motor themes of thematic analysis on root plasticity during drought and recovery research.

Basic themes	Occurrences	Motor themes	Occurrences
Drought	172	Water stress	75
Recovery	86	Stomatal conductance	33
Photosynthesis	68	Abscisic acid	22
Root	50	Chlorophyll fluorescence	22
Climate change	36	Biomass	20
Growth	31	Proline	19
Oxidative stress	18	Hydraulic conductivity	15
Nitrogen	17	Conductance	14
Resilience	16	Potential	13
Salt	16	Soil moisture	13

We expect basic themes, like root, recovery, climate change, and their relationship to yield and leaf traits, will drive future studies. The challenges of root and rhizosphere dynamic measurement will require the deployment of innovative technologies to accelerate root science. New technologies and methods, like noninvasive root and rhizosphere phenotyping, will be key to understanding root dynamics during drought and rewatering (Wasson et al., 2020). Mathematical modeling will also be important to simulate mechanisms of water transport as well as the discovery of plant traits for greater crop resilience and faster recovery (Hall, 1982; Carminati and Javaux, 2020; Maurel and Nacry, 2020; Javaux and Carminati, 2021; Joshi et al., 2022). Additionally, understanding the interactions with other abiotic stresses will be crucial in the context of “climate change” and “yield”.

### 3.3 Marked sources and scientific mapping of the outputs

#### 3.3.1 Trends in root plasticity during both drought and recovery

**Figure 5:**
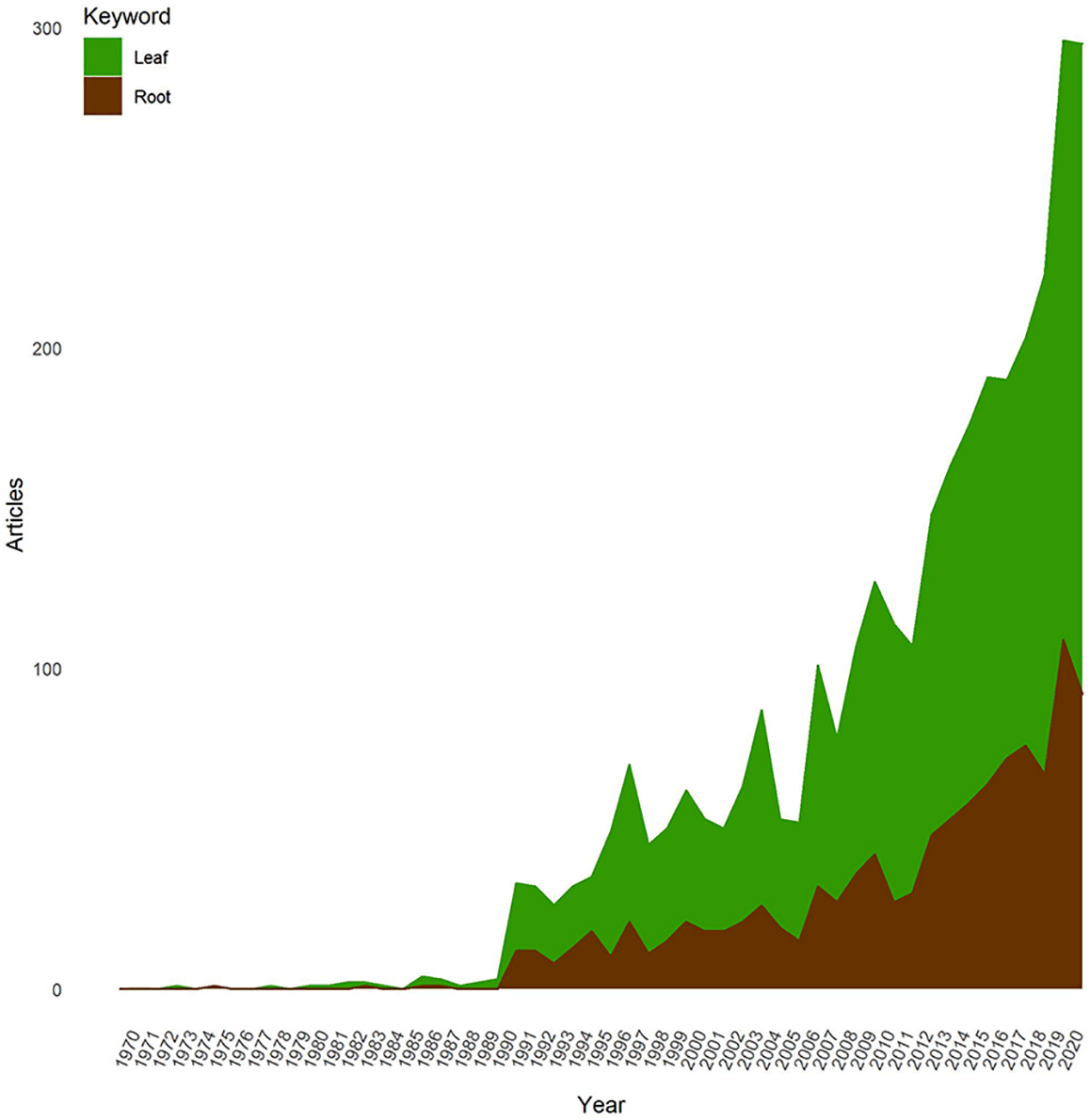
Temporal evolution of outputs on the shoot/root plasticity during drought and recovery research from 1900 to 2021.

Over the past 120 years, the number of publications on “root plasticity during drought and recovery” increased strongly, but still, the topic seems to be underdeveloped compared to shoot-related research (**Figure 5**). We distinguish three periods: 1900 to 1990, 1991 to 2004, and 2005 to 2022. For the first period, only 4 publications were found (in 1975, 1983, 1986, and 1988). During this period, few publications were uploaded to WOS and most researchers focused on drought but not recovery. The number of publications during that period was low in all sciences, but especially so in root research as measuring roots was challenging and few technologies were available. Thus, 99% of the analyzed publications were published in the last three decades with an annual increase of 7.20%/a, greater than the annual growth rate in Life Sciences of 5% (Bornmann et al., 2021). The results also revealed that the publications number improved very slightly from 1991-2006 with around 15 papers each year, while they dramatically increased during 2015-2022 (**Figure 5**) with 80% of publications found after 2005. The trend of root plasticity during both drought and recovery research was consistent with the trend in the related emerging topic of “carbon exchange in global drylands” (Liu et al., 2021).

#### 3.3.2 Marked countries and dominant institutes

USA and China were the most productive and cited countries. Among the 10 countries with the highest number of publications, the USA (relative frequency=71%) and China (69%) had a similar frequency and were 3 times higher than the third most productive country. Spain, Brazil, and Australia, at position 3-5, had a similar frequency of around 200 (**Table 3**). Although the most productive are also the most cited countries, USA-based publications were cited 2.2 times more often than those from China, with 14 *vs* 6 citations per publication. Spain, France, Australia, Italy, India, and Germany were positioned at 3-8 with 12-13 citations per article (**Table 3**). Three reasons may explain these ranking patterns: all these countries 1) have arid regions with severe drought stress; 2) have advanced technology for root research; 3) have many researchers and a higher GDP that ensures enough human and material resources for related research.

**Table 3:** Top 10 most productive and cited countries and most productive institutes with the publications of root plasticity during drought and recovery research during the period of 1900–2021.

Country production		Most cited countries		Most productive institutes	
Country	Number of publications	Country	Total Citations (Citation/Publication)	Affiliations	Articles
USA	772	America	10862 (14)	NORTHWEST A&F UNIV	44
China	745	China	4830 (6)	UNIV CALIF DAVIS	40
Spain	230	Spain	2835 (12)	CHINA AGR UNIV	34
Brazil	204	France	2595 (13)	KHON KAEN UNIV	31
Australia	180	Australia	2229 (12)	UNIV WESTERN AUSTRALIA	29
Germany	180	Italy	1770 (10)	GUANGXI UNIV	27
France	133	India	1137 (9)	TEXAS A&M UNIV	23
Italy	129	Germany	1135 (9)	UNIV CALIF LOS ANGELES	23
Japan	128	Brazil	1017 (8)	COLORADO STATE UNIV	22
India	123	Canda	895 (7)	UNIV FLORIDA	21

The collaboration map shows a similar ranking: USA (164), China (136), Germany (105), Spain (87), and Australia (74) had the highest collaboration frequency of all countries. Even though Germany’s ranking in the most productive and cited countries is not high, it still ranked in the third position according to the collaboration frequency, which indicates that Germany had many collaborations with other countries but less direct ownership in the topic. Germany is well known for its development of technologies, root phenotyping and soil-plant hydrology and as such is a looked-for partner, even though its agriculture is less threatened by drought compared to the other listed countries (**Figure S1**).

Our results showed that 1429 institutions all over the world have participated in root plasticity during both drought and recovery. The top ten most productive institutions contributed 27% (294 publications) of the total publications (**Tables 1**, **3**). From 1975 to 2022, the Northwest A&F University in China ranked first with the most publications (48), followed by the University of California, Davis (USA), China Agriculture University (China), Khon Kaen University (Thailand), and University of Western Australia (Australia). Consistent with the most productive and cited countries, 9 of the top 10 institutions belong to China, USA, or Australia. Khon Kaen University in Thailand stood out, as Thailand did not rank high in the country ranking.

Some highly productive authors affiliated with Khon Kaen University are A. Patanothai and S. Jogloy. For example, they concluded that during drought and recovery, peanut pod yield was associated with increased root surface area deeper in the soil (Jongrungklang et al., 2012; Jongrungklang et al., 2014). This is the only study we found that proposed a root ideotype for both drought and recovery, although there are other ideotype studies that focused on drought only.

#### 3.3.3 Competitive journals and top-cited publications

Studies on root plasticity during both drought and recovery were published in 334 journals, most related to botany or agronomy (**Table 1**). The top ten most productive and cited journals focus on plant research across scales from molecules to ecology. Plant and Soil, Tree Physiology, Frontiers in Plant Science, Agricultural Water Management, and Journal of Plant Physiology were found to be the five most prominent journals, with a note that Frontiers in Plant science is a relatively new journal. Tree Physiology, Plant Cell and Environment, Journal of Experimental Botany, New Phytologist, and Plant and Soil were the five highest topic-h-index journals (**Table 4**). In general, the h-index seems low, indicating that the topic is not receiving much attention despite its societal relevance. Plant Physiology, Tree Physiology, Journal of Experimental Botany, Plant Cell and Environment, and New Phytologist were the five journals that scored highest in total number citations, indicating the greater interest of publications in these journals as a source of bibliographic consultations.

**Table 4:** Top 10 most productive authors and journals with the publications of root plasticity during drought and recovery research during the period of 1900–2021.

Sources	Articles	h-index	Total citation
PLANT AND SOIL	37	17	801
TREE PHYSIOLOGY	36	24	1748
FRONTIERS IN PLANT SCIENCE	32	15	713
AGRICULTURAL WATER MANAGEMENT	24	14	637
JOURNAL OF PLANT PHYSIOLOGY	23	16	898
PLANT CELL AND ENVIRONMENT	22	20	1384
JOURNAL OF EXPERIMENTAL BOTANY	21	17	1479
NEW PHYTOLOGIST	21	17	925
ENVIRONMENTAL AND EXPERIMENTAL BOTANY	20	12	455
PLANT PHYSIOLOGY	18	16	2461

Eight of the top ten journals have a relatively broad scope in plant sciences, except for Tree Physiology and Agricultural Water Management, which have a strong focus on trees and crop water management, respectively (**Table 4**). Because drought stress is closely related to soil water content, drought events are easy to appear in a natural ecosystem like a forest ecosystem, so it’s not hard to understand why “Plant and Soil” and “Tree Physiology” were the most productive journals.

#### 3.3.4 Top-cited publications

In the SCI-E database, 1086 publications were found when searching across the last 120 years. The oldest publication was published in 1975 by Parrondo et al. entitled “Rubidium absorption by corn root tissue after a brief period of water stress and during recovery” published in Physiologia Plantarum, showing that reductions in rubidium uptake during a short period of water deficit only partially recovered during the post-recovery period (Parrondo et al., 1975). The effect of drought and rewetting on nutrient availability remains an important topic today. In the past, the focus was strongly on soil physical effects, such as reduced effective diffusion in dry soil (Hira and Singh, 1977). Recently a more complicated picture emerged involving the microbiota which influences phosphorus concentrations and sorption rates (Chen et al., 2021). Thereby, it still remains a question of how nutrient availability and uptake influences plant growth during drought and recovery and few papers deal with the question after Parrondo’s initial work. The study conducted by Xu et al. “Expression of a late embryogenesis abundant protein gene, HVA1, from barley confers tolerance to water deficit and salt stress in transgenic rice” (Xu et al., 1996), published in Plant Physiology in 1996, was the most cited article with 655 citations or 24 citations per year (**Table 5**). This study proved the important role of plant LEA (late embryogenesis abundant) proteins under drought and salt stress and its potentia1 for genetic crop improvement toward abiotic stress tolerance. Due to their versatility, LEA-related genes and their function have received much attention as potential drought and salt tolerance genes. Twenty six years later, LEA has been widely studied in the context of drought, still, its functioning is debated and real-world agronomic application is still a promise (Hernández-Sánchez et al., 2022). Among the top ten highly cited papers, two focused on genes (Xu et al., 1996; Swindell et al., 2007), one on proteins (Salekdeh et al., 2002), one on rhizosphere bacteria (Mayak et al., 2004), one on roots and branches hydraulic failure (Anderegg et al., 2012) and one on root-water transport modeling (Hsiao and Xu, 2000) (**Table 5**). Surprisingly, despite our root search term, there wasn’t any highly cited paper (>295) on root morphology or architecture. Root research on this topic of drought and recovery should get more attention in the future.

**Table 5:** Top 10 high cited papers with the publications of root plasticity during drought and recovery research during the period of 1900–2021.

Title of publications	Year of publications	Journal	Average citation per year	Total citations
Expression of a late embryogenesis abundant protein gene, HVA1, from barley confers tolerance to water deficit and salt stress in transgenic rice	1996	Plant Physiology	24.74	668
Adaptations of Endophyte-infected cool-season grasses to environmental stresses: Mechanisms of drought and mineral stress tolerance	2000	Crop Science	21.96	505
Plant growth-promoting bacteria that confer resistance to water stress in tomatoes and peppers	2004	Plant Science	25.63	487
General mechanisms of drought response and their application in drought resistance improvement in plants	2015	Cellular and Molecular Life Sciences	57.00	456
The roles of hydraulic and carbon stress in a widespread climate-induced forest die-off	2012	Proceedings of the National Academy of Sciences of the United States of America	41.09	452
Transcriptional profiling of Arabidopsis heat shock proteins and transcription factors reveals extensive overlap between heat and non-heat stress response pathways	2007	BMC Genomics	24.25	388
Development of drought-resistant cultivars using physio-morphological traits in rice	1995	Field Crops Research	13.14	368
Proteomic analysis of rice leaves during drought stress and recovery	2002	Proteomics	15.90	334
The crucial role of plant mitochondria in orchestrating drought tolerance	2009	Annals of Botany	22.29	312
Sensitivity of growth of roots versus leaves to water stress: biophysical analysis and relation to water transport	2000	Journal of Experimental Botany	12.83	295

## 4 Ideotypes and genetics

Ideotypes are an important way to identify and select better cultivars in agriculture, but so far we found only one publication that proposed a root ideotype for both drought and recovery. The study conducted by Jongrungklang et al. (2014) proved that greater root surface area of peanut at deeper soil layers contributed to a higher pod yield. As such, a shortlist of key root traits, and their genetics for a faster drought recovery remains speculation. In contrast, several studies have suggested root traits that are beneficial during drought. For example, Fonta et al. (2022) observed that a drought-tolerant rice line had, when exposed to drought, deeper and larger diameter roots. A field experiment carried out by Schneider et al. (2020) demonstrated that reduced root diameter of maize genotypes under drought can reduce the metabolic costs in soil exploration while penetration into drier soil is more difficult. The authors identified a gene locus (Zm00001d018342) which was also attributed a role in plasticity of root cross-sectional areas. Combining field and greenhouse experiments, Liao et al. (2022) demonstrated that under drought stress, high, and stable grain yield of “aus” rice varieties were positively related to “large-diameter” nodal roots, high and stable deep root growth. Genetically, qRT9 was associated with root thickness regulation (Li et al., 2015). Similarly, the region on chromosome 1 which is located near qDTY1.1, was associated with rice yield under drought conditions, also shoot and root plasticity responses in rice under drought stress, particularly increased deep rooting (Vikram et al., 2011; Wade et al., 2015; Sandhu et al., 2016). However, due to the complexity of drought recovery, root ideotypes may be highly context-specific. Here we suggest that by focusing on the drought period, science might have missed opportunities as there is no clarity if the traits that are proposed to be advantageous during drought also support a strong recovery. Recent studies of maize lines by Chen et al. (2016) and Gonzalez-Hernandez et al. (2021) emphasize that the recovery phase influenced the final plant biomass more than the drought phase. In the context of recovery after drought, many of the traits that have been suggested to enhance growth during drought, still need testing during the recovery period.

We further ask if genetic engineering can improve drought adaptability. Despite significant effort, surprisingly, only one transgenic cultivar (namely Monsanto’s DroughtGard) has been released to farmers so far (Passioura, 2020). The major reasons for the slow progress in the transgenic crop are the complexity of the drought environment, which often results in the lack of clear identification of the target environment, and also due to too much attention being assigned to a single drought process in the laboratory research. While under natural conditions the repeatable drought-rewetting cycle interacts with other (a)biotic factors, these were often ignored in research. Further understanding of root dynamics and the role of roots in plant resilience to both drought and recovery, therefore, should be taken into consideration in the future.

## 5 Implications and future perspectives

The continued fast growth of “root plasticity during drought recovery” has illustrated the status and importance of this field. We expect this topic to become more prominent in the near future, because of climate change, population growth, and the great need for a stable food supply. Understanding root plasticity during drought recovery is fundamental to increasing plant stress resilience and maximizing production on both the ecological and agricultural sides.

As the key element for plant water uptake and belowground process, how plant-environment interaction like plant hydraulic failure, above-/below-ground biomass allocation, plant/microbiology interaction, and species composition are affected under drought- recovery cycles context should be given more attention in the future. Drought recovery studies will be more helpful in understanding and predicting these processes.

During a drought, growth is reduced and a multitude of other physiological and phenotypic changes occur. Some are easily and quickly restored by rewatering, such as stomatal opening after closure, and others might be permanent “damage”. We summarized the current progress on plant and root plasticity during drought and recovery research, even though recovery related research is relatively rare (**Table 6**). During the recovery phase growth can be accelerated with relative growth rates that are greater than that of the control plants (Xu et al., 2009), but this is not always observed (Steinemann et al., 2015). Besides a rebalanced hydraulics, plants need to alter root traits, including root morphology/architecture, root anatomy, root exudate and rhizosphere microbiomes, to compensate for the reduced or abandoned plant growth during drought, and acclimate to the new soil environment. From the few reports that we found, root plasticity responses to drought recovery were highly species and scenario specific, making it difficult to generalize (**Table 6**). We hypothesize that the rate of the recovery depends on the performance of the root system. Root performance is influenced by 1) growth substrate condition: soil nutrient (N/P/K) content and soil structure; 2) drought intensity and frequency; 3) species: tree, grass, and crop; 4) growth stage e.g. early *vs* late season; 5) root physiology traits like ABA, water-soluble carbohydrates, nutrient homeostasis; 6) plasticity of root morphology and anatomy traits; 7) root and microbiome interaction, rhizosphere stability. Restoring root functioning, not in the least soil nutrient uptake, through restoration of root growth, root morphology, and rhizosphere functioning may be the key to fast whole plant recovery after a drought.

**Table 6:** Overview of root plasticity during drought and recovery research.

Phases	No drought	Moderate/extreme drought	Recovery
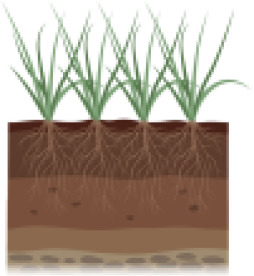 Whole plant	Plants grow actively	Plant photosynthesis/growth down-regulated or even stopped ^(1),(2)^, increased root: shoot ratio ^(3)^, more carbon allocated to roots ^(4)^	Plant photosynthesis/growth resumes or is even stimulated ^(1),(2),(3)^
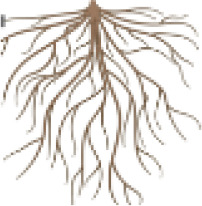 Root morphology/architecture	Root normal growth and distribution	Increased root growth at depth ^(5)^, “large-diameter” nodal roots, deep root angle ^(6)^, varied specific root length ^(7)^	Increased root biomass ^(8)^, decrease root growth rate at deep layer ^(9)^
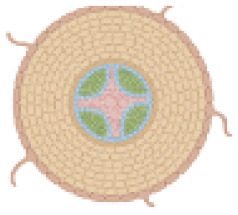 Root anatomy	Normal root anatomy	Fewer but larger cortical cells, higher root cortical aerenchyma, small xylem vessel area ^(10)^	Unknown
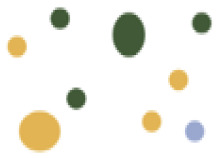 Root exudate	Normal root exudation	Down ^(11)^ or up-regulated root exudation ^(12),(13)^	Altered root exudation ^(11)^
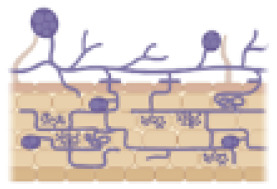 Rhizosphere microbiomes	Plant allocates carbon to rhizosphere bacteria and fungi	Less carbon allocated, changed microbiome composition, reduced heterotrophic microbiome activity or even stopped ^(14),(15)^	Increased activity, altered microbiome composition, and plant-microbe interaction ^(14),(15)^

(1) (Xu et al., 2009); (2) (Chen et al., 2016); (3) (Delfin et al., 2021); (4) (He et al., 2022); (5) (Schneider et al., 2020); (6) (Liao et al., 2022); (7) (Chandregowda et al., 2022); (8) (Slette et al., 2022); (9) (Jongrungklang et al., 2014); (10) (De Bauw et al., 2019); (11) (de Vries et al., 2019); (12) (Brunn et al., 2022); (13) (Preece and Penuelas, 2016); (14) (de Vries et al., 2020); (15) (Santos-Medellin et al., 2021).

## 6 Concluding remarks

We analyzed the scientific literature on root plasticity during drought and recovery in the past 120 years using bibliometric analysis on the premise that 1) the recovery phase is important as not all droughts are terminal and 2) roots and their responses to drought and rewatering are key to the resilience of both cropping systems and natural vegetations. The rewatering phase received much less attention than the drought periods and the root received much less attention compared to shoots. Aboveground physiological traits of model plants Arabidopsis, crop plants wheat, maize, as well as trees were found to be the most popular study areas. Co-occurrence network analysis showed that three clusters were classified for the keywords including photosynthesis response, physiological traits tolerance, and root hydraulic transport. Further, thematic evolution analysis showed a transition from classical agricultural and ecological research *via* physiological and molecular response, to root plasticity responding to drought recovery in recent years. Overall, both results showed that root plasticity’s role during drought and recovery is less focused. While progress has been made on leaf traits and root physiology areas, more attention should be given to root morphology and microbiome side using novel root phenotyping methods and mathematical modeling ways, to further understand root plasticity during both drought and recovery.

## Data availability statement

The original contributions presented in the study are included in the article/**Supplementary Material**. Further inquiries can be directed to the corresponding authors.

## Author contributions

CZ and JP conceived and designed the experiments and performed the analysis. CZ and JP wrote the first draft; other authors provided reviewing and editing advice. All authors contributed to the article and approved the submitted version.
