# Supplementary material for: Plant root plasticity during drought and recovery: What do we know and where to go?
Source: Front Plant Sci. 2023 Mar 16;14:1084355. doi: 10.3389/fpls.2023.1084355 (PMC10061088; doi:10.3389/fpls.2023.1084355)
Supplement: Supplementary file 1 [file DataSheet_1.pdf]

Supporting Information

Article title: Plant root plasticity during drought and recovery: What do we know and where to go?

Article acceptance date: 28 February 2023

Authors: Zheng C, Bochmann H, Liu Z, Kant J, Schrey SD, Wojciechowski T and Postma JA

The following supporting information is available for this manuscript:

Fig. S1 Country collaboration of published articles on root plasticity during drought and recovery topics from 1900 to 2021. The lines between the two countries indicate the collaboration frequency between two countries, the thicker the line is, the higher the frequency.

Table S1 Top 10 high cited papers with the publications of root plasticity during drought and recovery research during the period of 1900–2021 from Web of Science and its availability in Scopus.

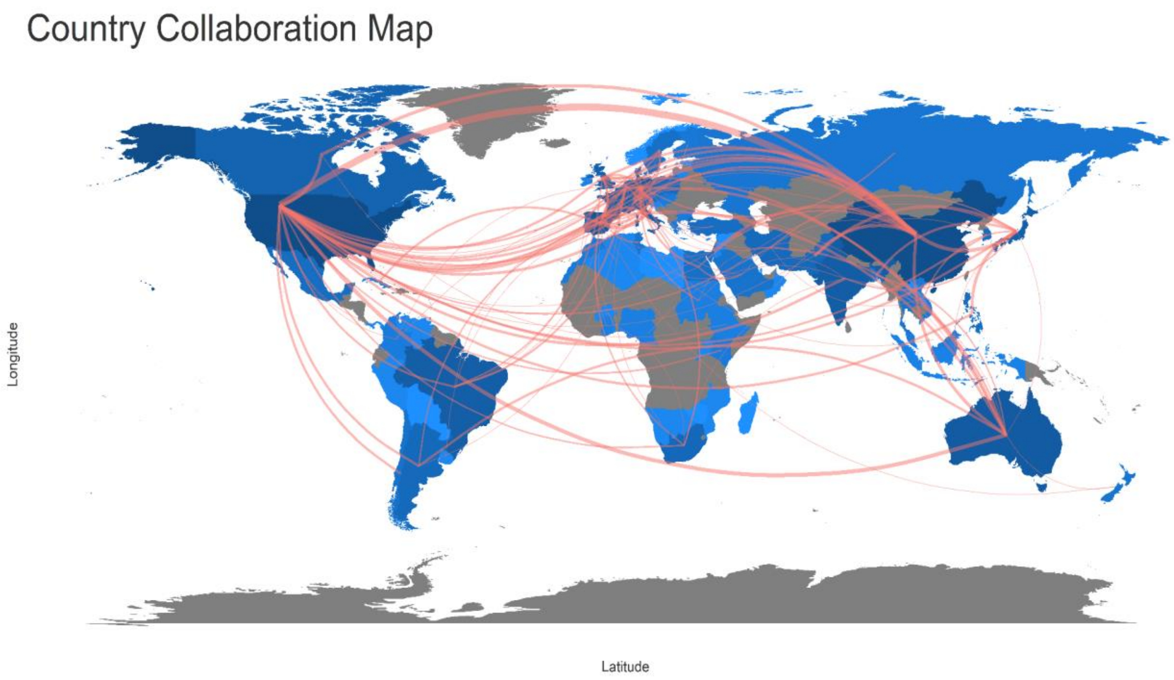

Fig. S1

Table S1

Title of publications	Year of publications	Journal	Total citation in Web of Science	**Availability in Scopus**	**Total citation in Scopus**
Expression of a late embryogenesis abundant protein gene, HVA1, from barley confers tolerance to water deficit and salt stress in transgenic rice	1996	Plant Physiology	668	Yes	804
Adaptations of Endophyte-infected cool-season grasses to environmental stresses: Mechanisms of drought and mineral stress tolerance	2000	Crop Science	505	Yes	588
Plant growth-promoting bacteria that confer resistance to water stress in tomatoes and peppers	2004	Plant Science	487	No	
General mechanisms of drought response and their application in drought resistance improvement in plants	2015	Cellular and Molecular Life Sciences	456	Yes	579
The roles of hydraulic and carbon stress in a widespread climate-induced forest die-off	2012	Proceedings of the National Academy of Sciences of the United States of America	452	No	

Transcriptional profiling of Arabidopsis heat shock proteins and transcription factors reveals extensive overlap between heat and non-heat stress response pathways	2007	BMC Genomics	388	Yes	425
Development of drought-resistant cultivars using physio-morphological traits in rice	1995	Field Crops Research	368	Yes	442
Proteomic analysis of rice leaves during drought stress and recovery	2002	Proteomics	334	No	
The crucial role of plant mitochondria in orchestrating drought tolerance	2009	Annals of Botany	312	Yes	353
Sensitivity of growth of roots versus leaves to water stress: biophysical analysis and relation to water transport	2000	Journal of Experimental Botany	295	Yes	355
